# Diagnostic Accuracy of Peritoneal Fluid GeneXpert in the Diagnosis of Intestinal Tuberculosis, Keeping Histopathology as the Gold Standard

**DOI:** 10.7759/cureus.3451

**Published:** 2018-10-15

**Authors:** Raheel Ahmad, Mehwish Changeez, Jahangir S Khan, Usman Qureshi, Maham Tariq, Sara Malik, Sheikh Haseeb Ahmad, Muhammad Salman Shafique

**Affiliations:** 1 Surgery, Holy Family Hospital, Rawalpindi, PAK

**Keywords:** abdominal tuberculosis, genexpert

## Abstract

Background

The diagnosis of abdominal tuberculosis is a major health challenge. Limited data are available to support the use of GeneXpert MTB/RIF in the diagnosis of abdominal tuberculosis. The current study is an analysis of the sensitivity and specificity of GeneXpert MTB/RIF for the diagnosis of abdominal tuberculosis, keeping histopathology as the gold standard.

Materials and methods

A prospective study was conducted in Surgery Unit-I of Holy Family hospital in the year 2017. Data of 21 patients presenting with abdominal tuberculosis were collected. The samples collected were ascitic fluid for GeneXpert and acid-fast bacilli (AFB) and a tissue sample for histopathology, which included either the enlarged lymph nodes or the involved gut segment.

Results

Out of a total of 21 patients, 10 were male and 11 were female. The predominant age group was less than 30 years with 76.2% cases. Of the 21 samples analyzed, all were positive for tuberculosis (TB) by histopathology. GeneXpert was positive in six and negative in 15 patients. The sensitivity of GeneXpert was 28.57% and specificity was 0%. The positive predictive value was 100%. The diagnostic accuracy was found to be 28.57%.

Conclusion

In our study, GeneXpert has shown poor sensitivity and specificity for the detection of abdominal TB from ascitic fluid samples. On the basis of this data, we lay stress on finding new tests and biomarkers for the rapid diagnosis of abdominal TB.

## Introduction

Tuberculosis is a significant health problem all over the world, especially in developing countries [1-2]. In 2013, nine million people became infected with tuberculosis and 1.5 million people died because of this disease all over the world [3]. Pakistan is ranked fifth among the high burden countries with tuberculosis with an annual 510,000 new cases [4]. A lack of typical clinical features led to a delay in diagnosis and the management of extrapulmonary tuberculosis [5].

Once the diagnosis has been considered, it is confirmed by using various tests. Culture is considered the gold standard for the diagnosis of Mycobacterium tuberculosis (MTB) but takes up to two to eight weeks. Microscopy for the detection of acid fast bacilli (AFB) is an inexpensive and rapid test but has low sensitivity and specificity and is also unable to differentiate between tuberculous and non-tuberculous mycobacterium (NTM). A histological examination of tissue is unable to differentiate tuberculosis from other diseases like sarcoidosis and NTM, however, they can detect stained tubercle bacilli. Other tests employed for the diagnosis of extrapulmonary tuberculosis (EPTB) include serological assays, Mantoux test, and polymerase chain reaction (PCR) assays. These tests vary in their sensitivity and specificity and some of these tests require relatively longer time [6-7].

The GeneXpert MTB/RIF system offers a rapid and efficient technique, which can detect Mycobacterium tuberculosis and drug resistance to rifampicin simultaneously within two hours [2]. The system is easy to use, carries the minimum risk of cross-contamination and is biosafe. World Health Organization (WHO) approved GeneXpert MTB/RIF in 2011 and recommended it for prompt implementation [8]. A recently published systemic review showed a pooled sensitivity of 88% and a pooled specificity of 98% [9] for the diagnosis of pulmonary tuberculosis. Another study assessed the convenience of an Xpert assay in EPTB and showed an 81.3% sensitivity and 99.8% specificity, considering culture and clinical diagnosis as the gold standard [10], however, still there is limited evidence to use GeneXpert in the diagnosis of EPTB [11].

The purpose of this study is to evaluate the sensitivity and specificity of GeneXpert MTB/RIF in the diagnosis of abdominal tuberculosis in comparison with histopathology as the standard with the aim of establishing the most appropriate laboratory tests algorithm on the basis of available knowledge and techniques.

## Materials and methods

A prospective study was conducted in Surgery Unit-I of Holy Family Hospital, where the data of 21 patients presenting with abdominal tuberculosis in the year 2017 was collected. A detailed history of clinical symptoms, a past history of tuberculosis, or the use of anti-tuberculous drugs and TB contact was taken. All patients presenting with a suspected complication of intestinal tuberculosis were investigated and managed by surgical intervention after initial resuscitation. Samples were collected from all patients undergoing laparotomy and operative findings were noted. The samples collected were ascitic fluid for GeneXpert and AFB and tissue samples for histopathology, which included either the enlarged lymph nodes or the involved gut segment.

Data were recorded and analyzed using Statistical Package for Social Sciences (SPSS) v20.0 (SPSS Inc., Chicago, IL, USA). For categorical variables, frequencies and percentages were reported. Sensitivity and specificity were calculated for GeneXpert, keeping histopathology as the standard.

True positive (TP): This was labeled if peritoneal fluid GeneXpert was positive and histopathology was also positive.

False positive (FP): This was labeled if peritoneal fluid GeneXpert was positive but histopathology was negative.

True negative (TN): This was labeled if peritoneal fluid GeneXpert was negative and histopathology was also negative.

False negative (FN): This was labeled if peritoneal fluid GeneXpert was negative but histopathology was positive.

## Results

A total of 21 patients were included in our study out of which 10 were male and 11 were female. The predominant age group was less than 30 years with 76.2% cases (Table 1). The most common presenting symptoms were abdominal pain, constipation, and vomiting in 38.1% patients. Constitutional symptoms of weight loss and fever were seen in 47.6% patients. A history of pulmonary tuberculosis and the use of anti-tubercular treatment (ATT) was positive in 57.1% and 47.6% patients, respectively.

**Table 1:** Age groups

Age	Frequency (n=21)	Percentage
Less than 30 years	16	76.2
more than 30 years	5	28.6

The most common preoperative finding was intestinal perforation with intestinal mass formation (33.3%) followed by intestinal perforation and stricture formation as shown in Table 2.

**Table 2:** Per op findings

Per op findings	Frequency (n=21)	Percentage
Intestinal perforation	5	23.8
Mass	4	19
Stricture	5	23.8
Intestinal perforation with mass formation	7	33.3

Resection and stoma formation was the most commonly performed procedure(42.9%) as seen in Table 3. 

**Table 3:** Surgical procedures performed

Surgical procedure	Frequency (n=21)	Percentage
Resection and stoma formation	9	42.9
Stoma formation	6	28.6
Stricturoplasty	3	14.3
Biopsy only	3	14.3

Of the 21 samples analyzed, all were positive for TB by histopathology. GeneXpert gave six positive and 15 negative results. However, for six cases, both histopathology and GeneXpert were positive. The sensitivity of GeneXpert was 28.57% and specificity was 0%. The positive predictive value was 100%. Diagnostic accuracy was found to be 28.57% (Tables 4-5).

**Table 4:** Results of GeneXpert

GeneXpert	Frequency (n=21)	Percentage
Positive	6	28.57
Negative	15	71.43

**Table 5:** Contingency table for GeneXpert

PERITONEAL FLUID GENEXPERT	Histopathology POSITIVE	Histopathology NEGATIVE	Total
Positive	6 (True positive)	0 (False positive)	6
Negative	15 (False negative)	0 (True negative)	15
Total	21	0	21

## Discussion

Pulmonary tuberculosis is the most common form of tuberculosis seen worldwide. Extrapulmonary tuberculosis involving lymph nodes, meninges, the intestine, bones, joints, genitourinary tract, etc. is also associated with significant morbidity and mortality. The abdomen is the sixth-most common site of extrapulmonary tuberculosis. The incidence of abdominal tuberculosis is increasing all over the world. However, very little knowledge and literature have been updated and it is still a diagnostic dilemma [12].

In 2013, WHO introduced the use of the GeneXpert MTB/RIF assay on pulmonary samples, where it has high sensitivity and specificity, and has thus been recommended for national TB programs in developing countries [1]. However, information regarding the performance of the MTB/RIF assay on extrapulmonary samples is still emerging. It has been granted as a conditional recommendation for the diagnosis of EPTB, however, the overall evidence has been cited as insufficient [13].

Previous studies of the MTB/RIF assay have reported a sensitivity of 100% for smear-positive respiratory and non-respiratory samples. Sensitivity for smear-negative samples was 57% and 37%, respectively [14]. Another study conducted by Zeka et al. showed an improved sensitivity of 100% for smear-positive extrapulmonary tuberculosis and 63% for smear-negative EPTB [15].

In our study, the sensitivity of GeneXpert for the diagnosis of abdominal tuberculosis was 28.57%. The positive predictive value and diagnostic accuracy were found be 100% and 28.57%, respectively. Similar low sensitivity on ascitic fluid samples (27.8%) was reported by the study conducted by Alvereza et al [16].

A study conducted by SB Rufai assessed the sensitivity of the MTB/RIF assay for the diagnosis of tuberculosis by using ascetic fluid samples, keeping MGIT-960 as the gold standard. Out of 67 patients, the MTB/RIF assay was positive in only 12 (17.9%) cases while 82.1% was negative. The study showed that the diagnostic yield of the MTN/RIF assay was low even in culture-positive specimens (70.5%), indicating that in highly proteinous body fluids, such as ascitic fluid, Xpert MTB/RIF‑negative cases must be investigated further using other phenotypic methods [1]. Xpert MTB/RIF has, however, a high positive predictive value (PPV), meaning that if GeneXpert is positive, the ATT can be started without waiting for other investigations.

A study conducted by Grant Theron on the determinants of PCR performance concluded that a low mycobacillary load in extrapulmonary samples as compared to pulmonary specimens is primarily responsible for the low sensitivity of the MTB/RIF assay in the diagnosis of extrapulmonary tuberculosis [13].

The low sensitivity of GeneXpert for the diagnosis of abdominal tuberculosis in our study can be attributed to the use of ascitic fluid as a specimen, small sample size, and poor sample-handling techniques.

## Conclusions

Even though the MTB/RIF assay has high sensitivity for the detection of pulmonary tuberculosis, in our study, GeneXpert evaluated for the detection of abdominal TB from ascitic fluid samples has shown poor sensitivity. On the basis of this data, therefore, we lay stress on finding new tools and discovering new biomarkers for the rapid diagnosis of abdominal TB.
